# Effects of Seasonal Variation on the Alkaloids of Different Ecotypes of *Epichloë* Endophyte-*Festuca sinensis* Associations

**DOI:** 10.3389/fmicb.2019.01695

**Published:** 2019-07-25

**Authors:** Weihu Lin, Yu Kuang, Jianjun Wang, Dongdong Duan, Wenbo Xu, Pei Tian, Clement Nzabanita, Meining Wang, Miaomiao Li, Bihua Ma

**Affiliations:** ^1^State Key Laboratory of Grassland Agro-ecosystems, Key Laboratory of Grassland Livestock Industry Innovation, Ministry of Agriculture and Rural Affairs, College of Pastoral Agriculture Science and Technology, Lanzhou University, Lanzhou, China; ^2^Agricultural and Rural Bureau of Liling City, Liling, China

**Keywords:** alkaloids, *Festuca sinensis*, *Epichloë* endophyte, ecotype, season

## Abstract

The *Epichloë* endophyte-*Festuca sinensis* association produces alkaloids which can protect the host plant from biotic and abiotic stresses. Alkaloid concentrations depend on the genetic predisposition of grass and endophyte, and are affected by the environment. Endophyte infected *F. sinensis* of six ecotypes were grown in experimental field and greenhouse for 2 years. Their aboveground plant tissues were collected each season to test for peramine, lolitrem B, and ergot concentrations. The results showed that seasonal changes affected the peramine, lolitrem B and ergot concentrations of *Epichloë* endophyte-*F. sinensis* associations; and these three different alkaloids responded differently to seasonal variation. The peramine concentration of six ecotypes of *F. sinensis* decreased significantly (*p* < 0.05) from spring to autumn. The lolitrem B concentration of *F. sinensis* was higher in autumn than in other seasons. Ergot concentrations of five ecotypes (41, 57, 84, 99, and 141) of *F. sinensis* peaked in the summer, and lowered in spring and autumn. In addition, the ecotype has insignificant effect (*p* > 0.05) on the peramine and lolitrem B concentrations of *F. sinensis*, but it has a significant impact (*p* < 0.05) on the ergot concentrations. We concluded that the seasonal variation and ecotypes can influence the alkaloids produced by the *F. sinensis-*endophyte associations, but the effects of seasonal conditions on the alkaloid concentrations are more pronounced than ecotypes.

## Introduction

In grassland ecosystems, most grasses are infected by endophytic fungi and produce alkaloids in their tissues (Nan, 1996a,b; Gao and Nan, 2007). Many grass species are symbiotic with systemic, vertically transmitted, asymptomatic *Epichloë* endophytic fungi. These fungi often generate alkaloids in order to defend the host against herbivores (Helander et al., 2016). Alkaloids increase the capacity of host plants to resist the biotic and abiotic stresses (Charles, 1993; Malinowski and Belesky, 2000). In the past few decades, scholars have explored the categories, mechanism of synthesis, and factors driving the synthesis of alkaloids. The alkaloids are the products of complex biochemical pathways which are becoming well understood (Schardl et al., 2012, 2013a). Until recently, four alkaloid categories have been identified, i.e., indole diterpenes, ergot alkaloids, peramine, and loline (Johnson et al., 2013). Alkaloid concentrations depend on the genetic predisposition of grass and endophyte, and are influenced by the environmental conditions, e.g., seasonal variation, plant age, ecotypes, plant nutrition, temperature change, and drought occurrence (Helander et al., 2016; Fuchs et al., 2017).

Endophytes are microorganisms that reside within sturdy plant tissues intercellularly and/or intracellularly but usually remain asymptomatic and do not show any noticeable damage to the host (Siegel et al., 1987; Nazir and Rahman, 2018). They are an important component that colonizes in healthy tissues of living plants and can be readily isolated from any microbial or plant growth medium. Endophytic fungal associations with grasses are very common, and the most intensively studied are those between ascomycete fungi and temperate grasses, in particular those involving asexual endophytes of the genus *Epichloë* (Schardl et al., 2004; Li et al., 2017). Negative impact on herbivores is attributed to secondary metabolites alkaloids, which are produced by the endophytic fungus-grass symbiosis (Schardl et al., 2004). Animal toxicity due to the accumulation of nitrogenous compounds, e.g., endophyte-derived alkaloids, particularly in areas and periods under abiotic stress, still prevents widespread application of endophyte-infected grasses in agroecosystems (Hume et al., 2016). Over 30 years of study on the benefits of symbiotic *Epichloë* fungal endophytes for host grasses, investigations have focused on the major agricultural species, tall fescue and perennial ryegrass. However, many other grass species remain to be evaluated for the effects of *Epichloë* endophytes.

*Festuca sinensis* is a key native cool-season perennial grass species that is widely distributed across the cold and semi-arid regions. It plays an important role in the meadow ecosystem of the Qinghai-Tibetan Plateau in China (Lin et al., 2018). This grass species often hosts the systemic endophytic fungus, *Epichloë* endophyte (Zhou et al., 2015b; Song et al., 2016). Recent studies have detected three different alkaloids (i.e., peramine, lolitrem B, and ergot) in *F. sinensis* due to the infection of *Epichloë* endophyte tissue (Zhou et al., 2015a; Tian et al., 2018). An earlier study found that the presence of *Epichloë* endophyte could promote the growth of *F. sinensis* (Nan and Li, 2004). Some recent studies reported that *Epichloë* endophyte could also enhance host plant resistance to drought and waterlogged conditions (Wang et al., 2017) and promote its seeds’ germination and growth (Peng et al., 2013). On top of the direct defense (in the form of alkaloids), the *Epichloë* endophyte enhances the host plant defense through improving the plant odor, which in turn attracts more olfactory foraging aphid predators (Fuchs and Krauss, 2018). Cold shock also induces ergot alkaloid changes in *F. sinensis* symbionts, and the degree of these changes differs between ecotypes and temperatures (Zhou et al., 2015a). Previous studies found that ecotypes can affect the concentrations of alkaloids in *Lolium perenne* and *Festuca pratensis* (Cagas et al., 1999). In addition, higher alkaloid levels were detected in *Achnatherum inebrians* plants under salt or drought stress and the concentrations of alkaloids increased over the plant growing period (Zhang et al., 2011). Interestingly, despite the infection of *Epichloë* endophyte on *F. sinensis*, there were no reports of intoxicated grazing animals on consuming *F. sinensis*-endophyte associations. This might indicate the existence of “mammalian-safe” nontoxic endophytes. Nonetheless, it remains unclear whether seasonal variation and ecotypes affect the alkaloid concentration of *Epichloë* endophyte-*F. sinensis* associations.

By performing a series of experiments under field and greenhouse conditions, we determine the alkaloid concentration of six ecotypes of *Epichloë* endophyte-*F. sinensis* associations under different seasonal conditions. We also explore the effects of seasonal variation, ecotypes, and their interactions on the alkaloids (peramine, lolitrem B, and ergot) produced by the *Epichloë* endophyte-*F. sinensis* associations.

## Materials and Methods

### Plant Materials and Growing Conditions

The seeds of six *F. sinensis* ecotypes were obtained from the Institute of Grassland, Qinghai Academy of Animal Husbandry and Veterinary Sciences and the Sichuan Academy of Grassland Science. These seeds of six locations were collected in summer 2013 (Table 1), regarded as six ecotypes, and 100 g seed of per ecotype was dispatched to Lanzhou University in February 2014. The storage temperature was maintained at 4°C to retain seed viability (Tian et al., 2018).

**Table 1:** Collection location of six *Epichloë* endophyte-*F. sinensis* association ecotypes.

Location	Ecotype	Latitude	Longitude	Altitude (m)
Bazanggou, Pingan, Qinghai, China	41	36°20′ N	102°06′ E	3,129 m
Bazanggou, Pingan, Qinghai, China	57	36°20′ N	102°06′ E	2,994 m
Shagou, Pingan, Qinghai, China	84	36°17′ N	102°05′ E	3,032 m
Guchengzhen, Pingan, Qinghai, China	99	36°17′ N	101°58′ E	3,060 m
Hongyuan, Sichuan, China	111	32°48′ N	102°33′ E	3,491 m
Shihuiyao, Pingan, Qinghai, China	141	36°19′ N	101°51′ E	2,922 m

Here, the first set (consisted of six different plant shoots) of ecotypes of *F. sinensis* were obtained from the field of the College of Pastoral Agriculture Science and Technology, Yuzhong campus of Lanzhou University (latitude: 35°89′ N, longitude: 104°39′ E; altitude: 1,653 m). These plants were grown for about 2 years (from June 2014 to December 2016). The second set (also consisted of six different plant shoots) were acquired from the greenhouse of Lanzhou University, and were grown for about 2 years (from April 2014 to December 2016). Note that the seed sources of these growing conditions (field and greenhouse) are identical.

### Experimental Design

The seeds of six *F. sinensis* ecotypes were planted in the two sets, to explore the effects of seasonal variation on the alkaloids of different ecotypes of *Epichloë* endophyte-*F. sinensis* associations in the same growing location (Lanzhou University).

In June 2014, the six different ecotypes of *F. sinensis* were planted under field conditions. There were five field pots for each ecotype. Each experimental field pot was 4 m long and 3.2 m wide, and the seedlings were planted at 40-cm intervals. Each pot contained five plants of each ecotype, which were randomly arranged. The E+ and E− plants of these six ecotypes of *Festuca sinensis* were set up in May 2014 after aniline blue microscopic examination (Li et al., 2004) in greenhouse and transplanted in field block with random design (Kuang, 2016; Tian et al., 2018).

In March 2014, healthy and well-filled seeds were planted into a 72-hole plastic seedling tray containing sterilized vermiculite under greenhouse conditions. One month after sowing, the presence of endophyte in the seedlings was determined by performing the microscopic examination of host leaf-sheath samples after staining with aniline blue. The selected plants E+ (infected by endophyte) were transported to the pots and were randomly placed in the greenhouse. They were maintained at a constant temperature (temperature: 24°C, moisture: 50%) and a 12:12-h light-dark cycle. Each ecotype of *F. sinensis* was transplanted into pot and the process was repeated 40 times.

Plant samples were obtained in March (spring), June (summer), September (autumn), and November (winter), respectively, in 2016. The sampling conducted four times a year, collected a total of 1 year. The plant shoots of six *F. sinensis* ecotypes were harvested by cutting them at 2 cm above the soil surface. For each ecotype under field conditions, we randomly sampled five E+ plants from five field pots. All plant samples were freeze-dried (PowerDry LL 3000; Thermo Fisher Scientific, Waltham, MA, USA). Subsequently, the plant samples were ground into powder in a mixer mill (MM 400; Retsch, Haan, Germany) for 2 min at 30 Hz for analyzing the endophyte-derived alkaloid content.

### Determination of Alkaloids

The ground shoot powder was weighed for measuring the concentrations of peramine, lolitrem B, and ergot *via* high-performance liquid chromatography (HPLC) (Gallagher et al., 1985; Barker et al., 1993; Ball et al., 1995; Tian et al., 2013, 2018). The “ergot” is total ergot alkaloid. Five biological replicates per ecotype and three technical replicates per biological replicate were tested (Tian et al., 2018).

#### Peramine Analysis

The 100-mg freeze-dried shoot powder was extracted in a solution of 3 ml of methanol and 3 ml of trichloromethane for 30 min under ultrasonic cleaner. The mixture material was centrifuged for 10 min at 1000 rpm, where 3 ml of *n*-hexane and 3 ml of ultra-pure water were added respectively, then extracted for 30 min and centrifuged for 12 min at 1000 rpm. Peramine was removed from shoot powder extracts by passing 1-ml portions through preconditioned 1-ml Agilent Bond Elut carboxylic acid (CBA) columns packed with 100 mg of adsorbent. The peramine was then eluted with 1 ml of a 5% formic acid-40% methanol solution. Peramine was measured by HPLC with an Agilent (Agilent 1100, America) liquid chromatograph fitted with a C18 column (Eclipse XDB-C18, 250 mm × 4.6 mm, 5 μm). Detection was performed with an ultraviolet (UV) wavelength spectrophotometric detector set at 280 nm. Mobile phase “A” was 1.8 g L^−1^ guanidine carbonate, and phase “B” was acetonitrile. The quantity of peramine in 25-μl injection samples was determined, based on pre-established standard curve peak area values. All reagents were chromatographically pure.

#### Lolitrem B Analysis

The 200-mg freeze-dried shoot powder was extracted in a solution of 4 ml of dichloromethane for 5 min under ultrasonic cleaner. The mixture material was centrifuged for 10 min at 1000 rpm. Lolitrem B was removed from shoot powder extracts by passing 2.5-ml portions through preconditioned Sep-Pak (Agilent Bont Elut SI, 500 mg, 3 ml) columns. The lolitrem B was then eluted with 0.7 ml of a 20% methanol-80% dichloromethane solution. Lolitrem B was measured by HPLC with an Agilent (Agilent 1100, America) liquid chromatograph fitted with a Zorbax-RX column (Zorbax RX-SIL, 250 mm × 4.6 mm, 5 μm). Detection was performed with a fluorescence detector, the excitation wavelength was set at 268 nm and the emission wavelength was 440 nm. Mobile phase “A” was acetonitrile, and phase “B” was dichloromethane. The quantity of lolitrem B in 20-μl injection samples was determined, based on pre-established standard curve peak area values. All reagents were chromatographically pure.

#### Ergot Analysis

The 200-mg freeze-dried shoot powder was extracted in a solution of 4-ml 20% glacial acetic acid for 5 min under ultrasonic cleaner. The mixture material was centrifuged for 5 min at 1700 rpm. Ergot was removed from shoot powder extracts by passing 2-ml portions through preconditioned PCX (Agilent Bond Elute, 60 mg, 3 ml) columns. The ergot was then eluted with l ml of a 95% methanol-5% ammonium hydroxide solution. Ergot was measured by HPLC with an Agilent (Agilent 1100, America) liquid chromatograph fitted with a C18 column (Eclipse XDB-C18, 250 mm × 4.6 mm, 5 μm). Detection was performed with a fluorescence detector, the excitation wavelength was set at 312 nm, and the emission wavelength was 427 nm. Mobile phase “A” was 7.708 g L^−1^ ammonium acetate, and phase “B” was acetonitrile. The quantity of lolitrem B in 20-μl injection samples was determined, based on pre-established standard curve peak area values. All reagents were chromatographically pure.

### Data Analysis

Statistical data analysis was performed with the SPSS Inc. (Released 2009. PASW Statistics for Windows, Version 18.0. Chicago: SPSS Inc). The effects of seasonal variation and ecotypes on the peramine, lolitrem B, and ergot concentrations were evaluated using one-way and two-way ANOVA, respectively. A repeated-measure ANOVA with Fisher’s least significant difference (LSD) test was applied to determine whether the differences between the means were statistically significant or not. Statistical significance was defined at the 95% confidence level. The means were expressed together with their standard errors.

## Results

### Peramine

**Figure 1:**
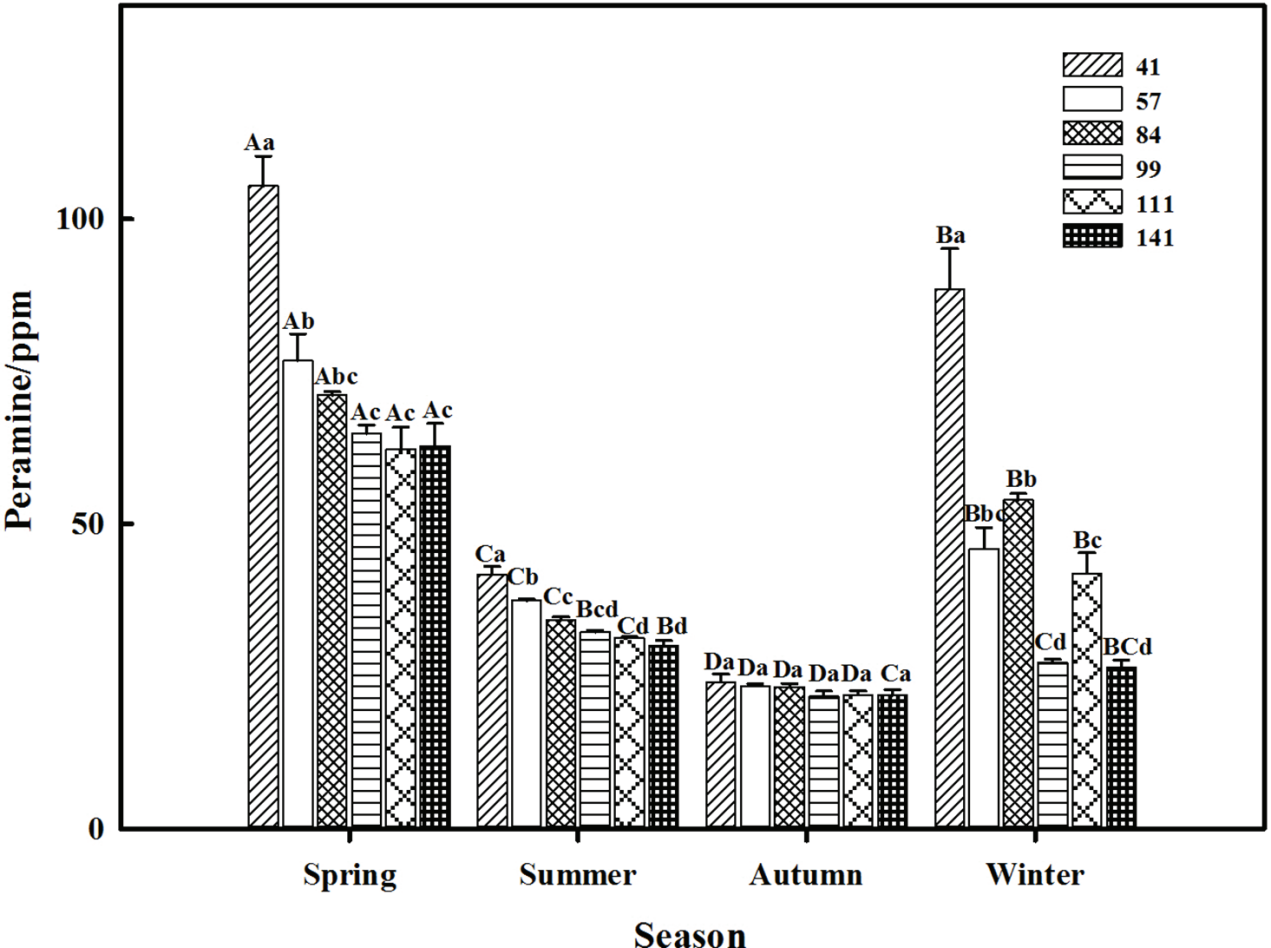
Effects of season and ecotype on the peramine concentration of *Epichloë* endophyte-*F. sinensis* under field conditions. Values are the mean ± standard error (SE). Different uppercase letters indicate significant differences among the same ecotype under different seasonal conditions at *p* < 0.05, different lowercase letters indicate significant differences among the different ecotypes under the same seasonal conditions at *p* < 0.05.

**Figure 2:**
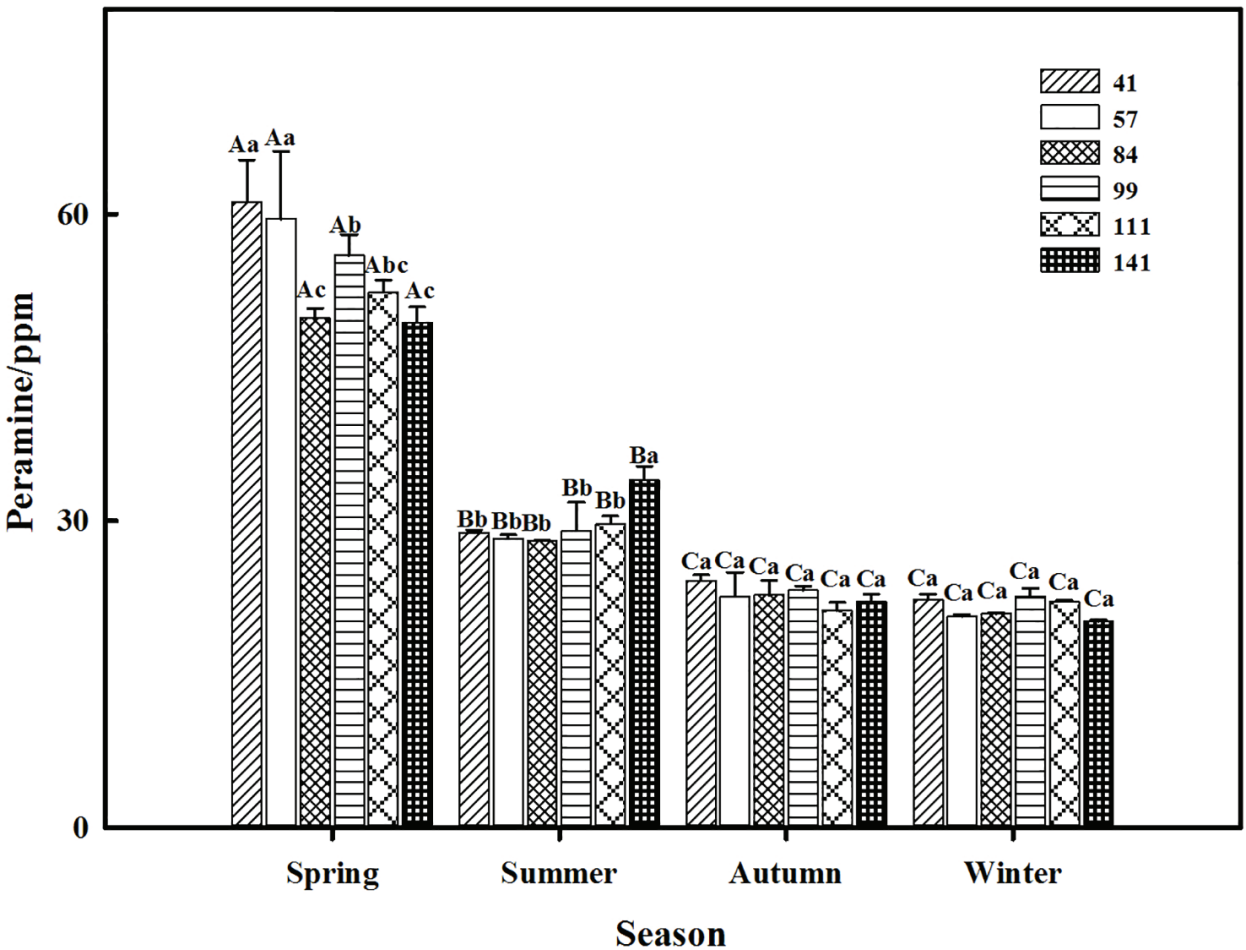
Effects of season and ecotype on the peramine concentration of *Epichloë* endophyte-*F. sinensis* under greenhouse conditions. Values are the mean ± standard error (SE). Different uppercase letters indicate significant differences among the same ecotype under different seasonal conditions at *p* < 0.05, different lowercase letters indicate significant differences among the different ecotypes under the same seasonal conditions at *p* < 0.05.

The two-way ANOVA revealed that seasonal variation significantly affected the peramine concentration of *F. sinensis* (*p* < 0.05), but the role of ecotype appeared statistically insignificant (Tables 2, 3). However, the interactions between ecotypes and seasonal variation had a significant impact on the peramine concentration of *F. sinensis* (*p* < 0.05; Table 3). The peramine concentrations of six ecotypes of *F. sinensis* decreased significantly (*p* < 0.05) from spring to autumn (Figures 1, 2), but they increased in winter only under field conditions (Figure 1). The peramine concentrations of six ecotypes of *F. sinensis* bottomed in autumn, and the differences between the ecotypes were insignificant (Figures 1, 2). The peramine concentrations of ecotype 41 and 141 reached the peak and trough during the trial period, respectively (Figures 1, 2).

**Table 2:** Results of two-way ANOVA for the effects of season and ecotype on peramine, lolitrem B, and ergot concentration of *Epichloë* endophyte-*F. sinensis* under field conditions.

Treatment (field)	df	Peramine		Lolitrem B		Ergot	
		*F*	*p*	*F*	*p*	*F*	*p*
Ecotype	5	0.8810	0.4540	0.4470	0.7200	0.2830	0.8380
Season	3	3.8900	0.0030	3.7900	0.0040	6.9410	<0.001
Ecotype × Season	15	1.6990	0.0640	1.8620	0.0370	4.2810	<0.001

**Table 3:** Results of two-way ANOVA for the effects of season and ecotype on peramine, lolitrem B, and ergot concentration of *Epichloë* endophyte-*F. sinensis* under greenhouse conditions.

Treatment (greenhouse)	df	Peramine		Lolitrem B		Ergot	
		*F*	*p*	*F*	*p*	*F*	*p*
Ecotype	5	1.8785	0.1157	0.9566	0.4537	5.0900	0.0010
Season	3	384.1653	<0.001	19.4855	<0.001	22.4080	<0.001
Ecotype × Season	15	2.1973	0.0200	2.9501	0.0023	2.9070	0.0030

### Lolitrem B

**Figure 3:**
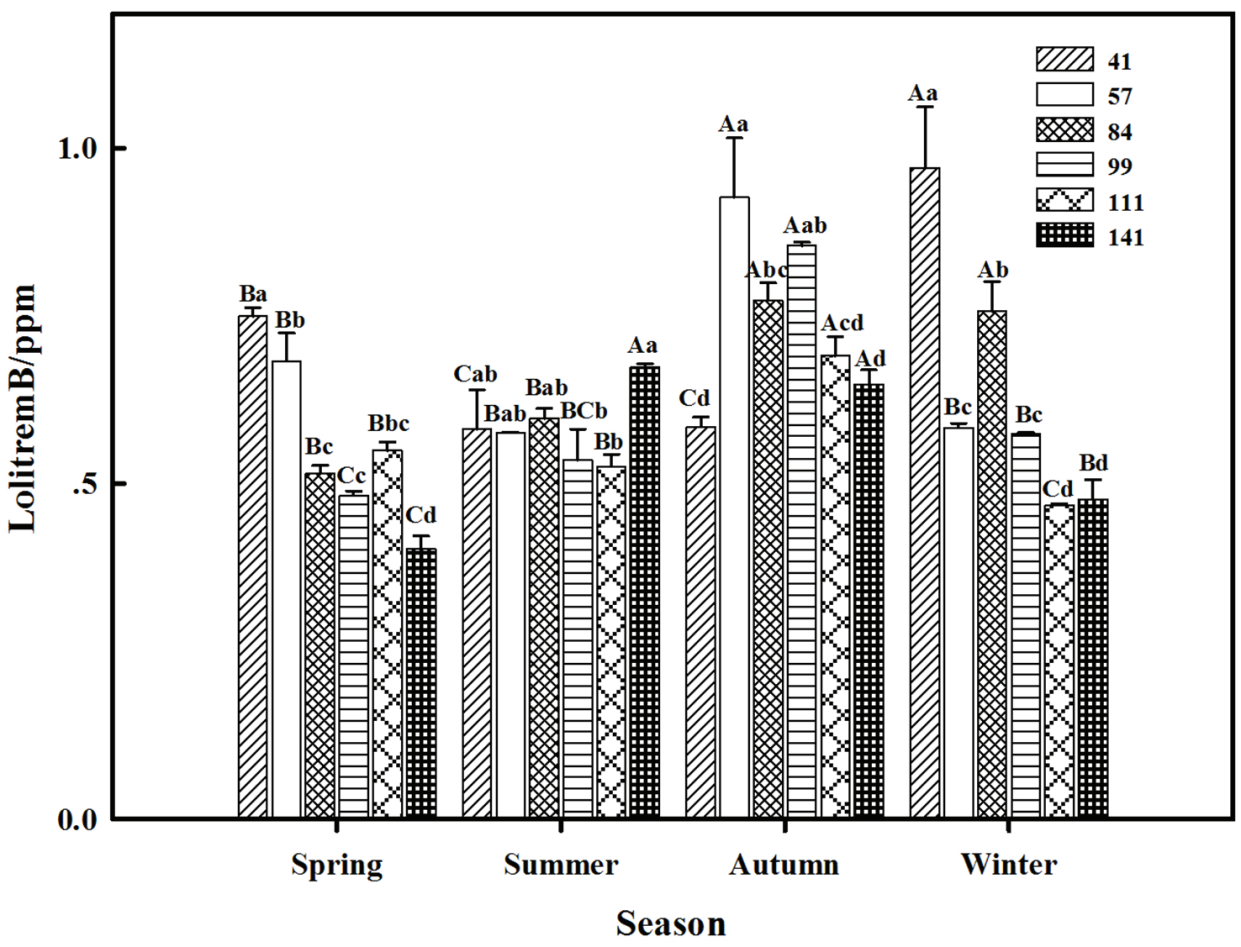
Effects of season and ecotype on the lolitrem B concentration of *Epichloë* endophyte-*F. sinensis* under field conditions. Values are the mean ± standard error (SE). Different uppercase letters indicate significant differences among the same ecotype under different seasonal conditions at *p* < 0.05, and different lowercase letters indicate significant differences among the different ecotypes under the same seasonal conditions at *p* < 0.05.

**Figure 4:**
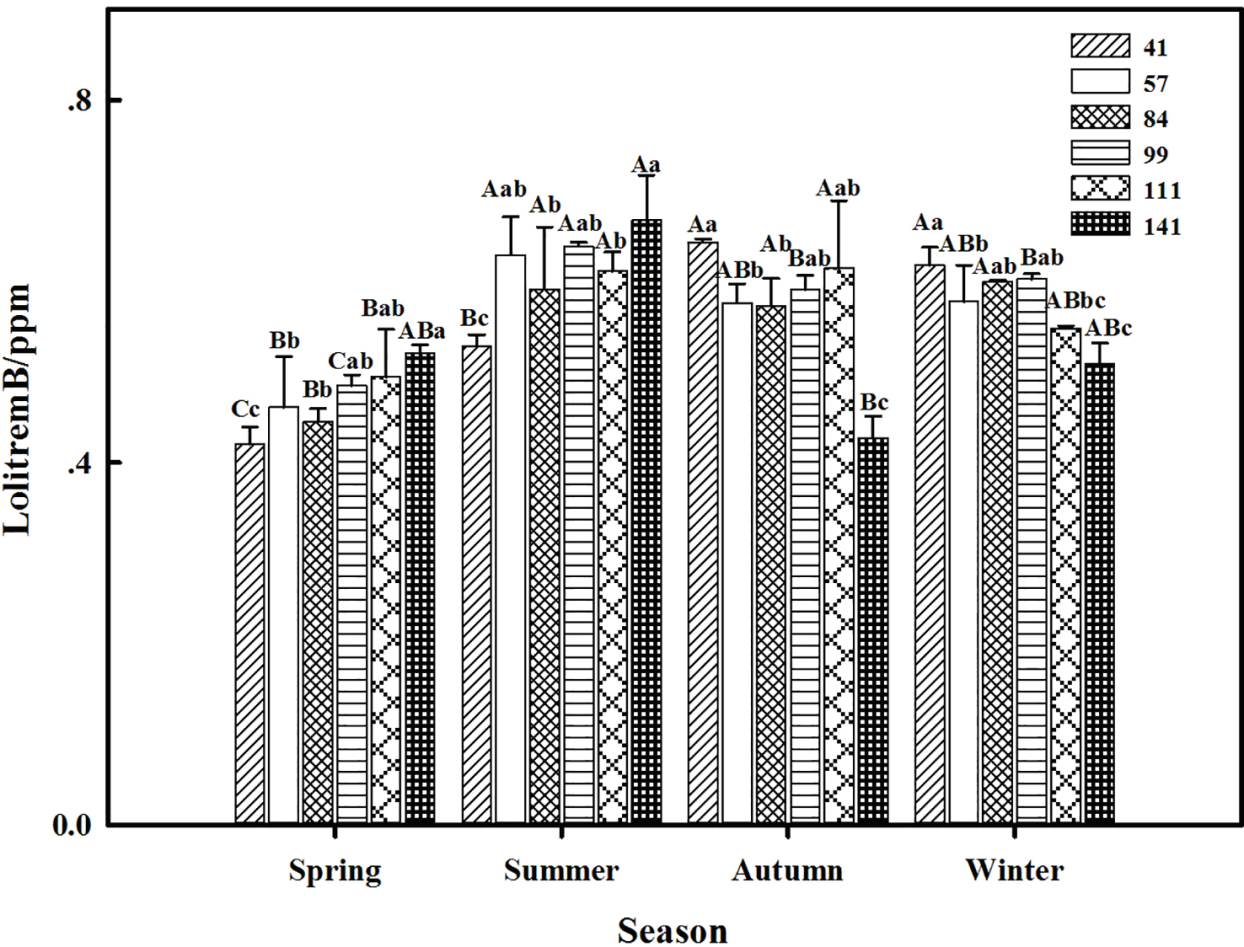
Effects of season and ecotype on the lolitrem B concentration of *Epichloë* endophyte-*F. sinensis* under greenhouse conditions. Values are the mean ± standard error (SE). Different uppercase letters indicate significant differences among the same ecotype under different seasonal conditions at *p* < 0.05, and different lowercase letters indicate significant differences among the different ecotypes under the same seasonal conditions at *p* < 0.05.

The results showed that seasonal variation significantly affected the lolitrem B concentration of *F. sinensis* (*p* < 0.05). Although the ecotype did not significantly affect it, the interactions between ecotype and seasonal variation were obvious (*p* < 0.05) on the lolitrem B concentration of *F. sinensis* (Tables 2, 3). Under field conditions, the lolitrem B concentration of ecotype 41 of *F. sinensis* was significantly higher in spring-winter (*p* < 0.05) than in summer-autumn (Figure 3). Under greenhouse conditions, the lolitrem B concentration of ecotype 41 of *F. sinensis* was significantly higher in autumn-winter than in spring-summer (Figure 4). While the lolitrem B concentration of ecotype 57 of *F. sinensis* was significantly higher in autumn (*p* < 0.05) than other seasons under field conditions (Figure 3), its concentration hit the lowest in spring with little difference among other seasons under greenhouse conditions (Figure 4). The lolitrem B concentrations of ecotype 84, 99, 111, and 141 of *F. sinensis* were significantly higher in autumn (*p* < 0.05) than in spring under both field and greenhouse conditions (Figures 3, 4). Overall, the lolitrem B concentration of *F. sinensis* was higher in autumn than in other seasons.

### Ergot

**Figure 5:**
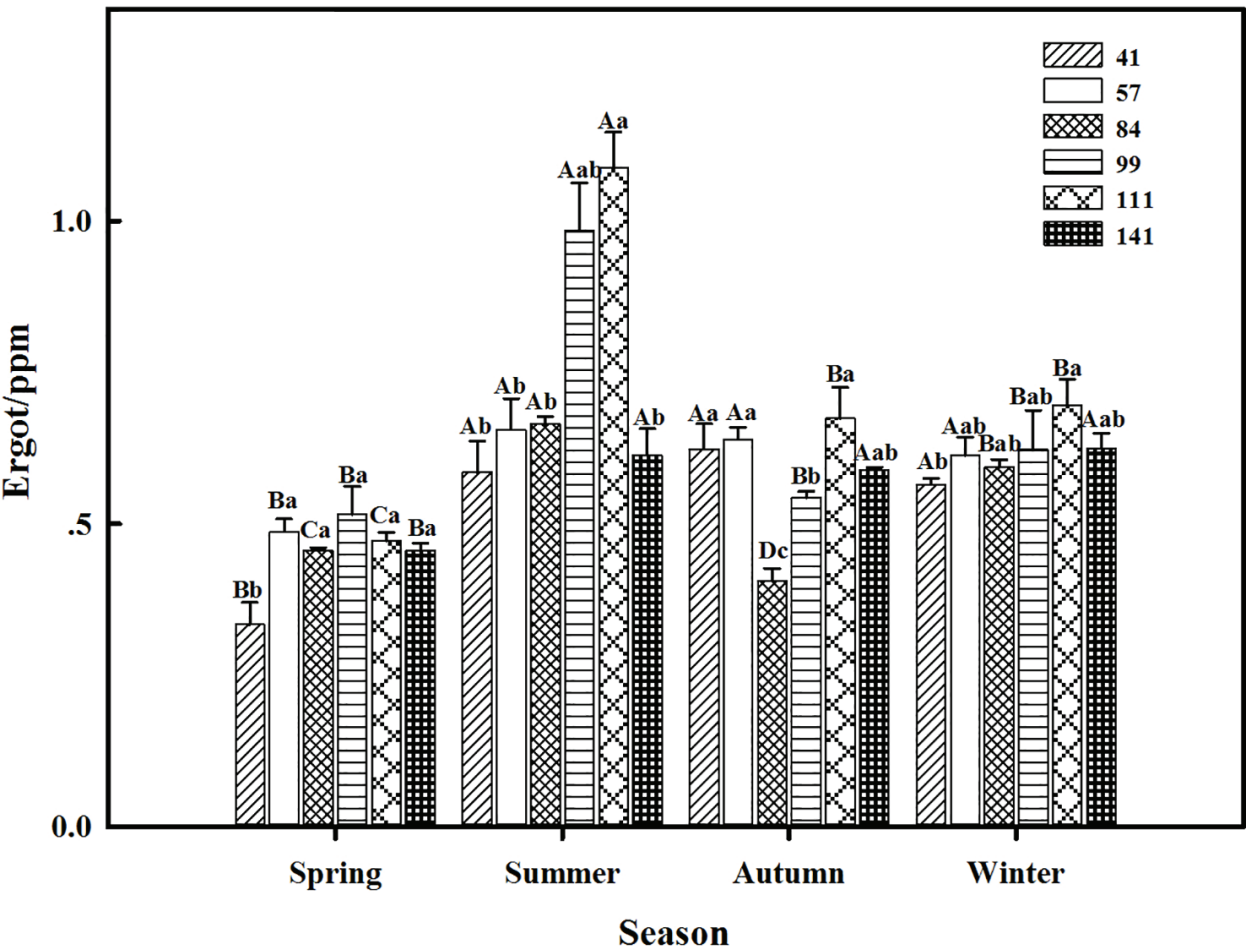
Effects of season and ecotype on the ergot concentration of *Epichloë* endophyte-*F. sinensis* under field conditions. Values are the mean ± standard error (SE). Different uppercase letters indicate significant differences among the same ecotype under different seasonal conditions at *p* < 0.05, and different lowercase letters indicate significant differences among the different ecotypes under the same seasonal conditions at *p* < 0.05.

**Figure 6:**
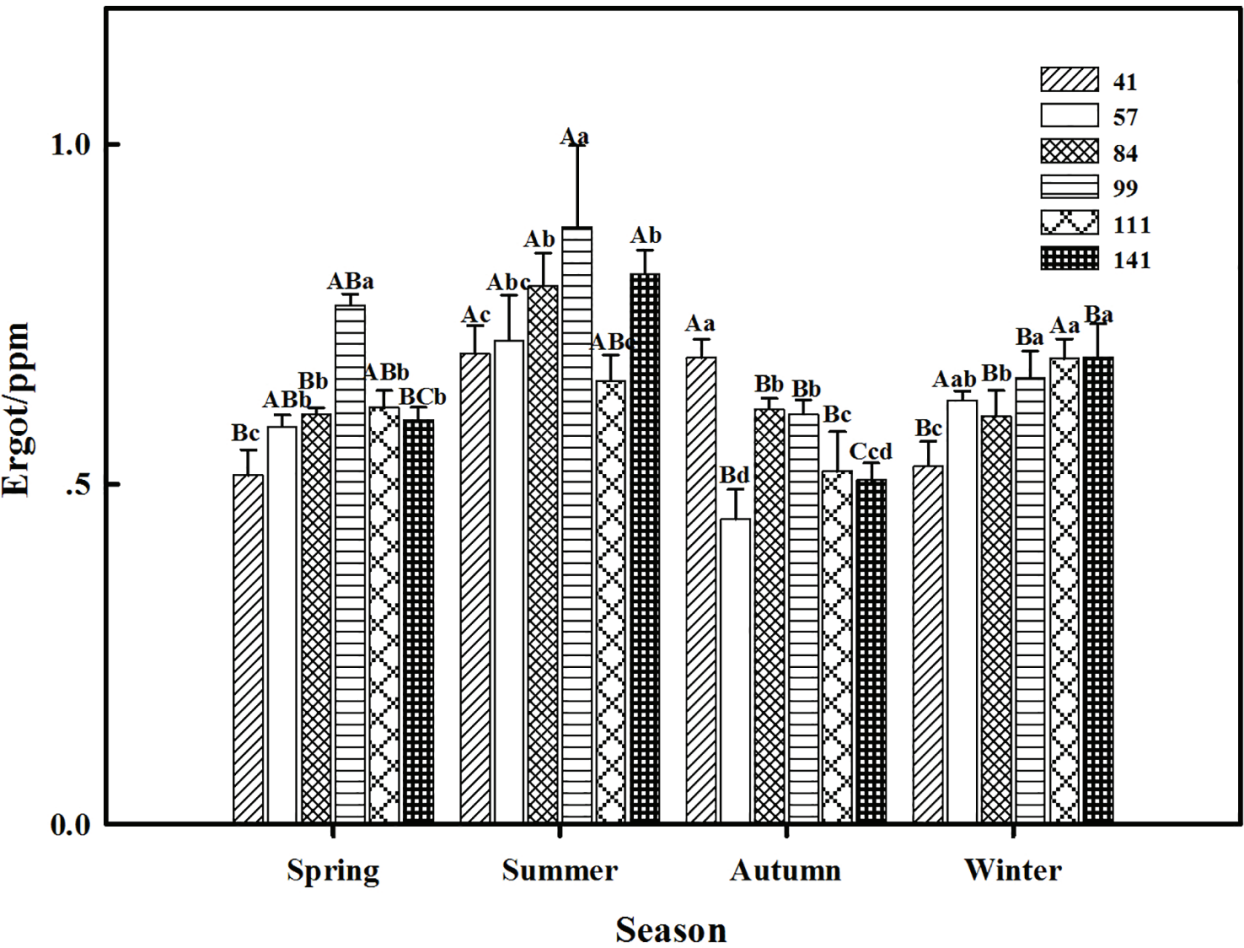
Effects of season and ecotype on the ergot concentration of *Epichloë* endophyte-*F. sinensis* under greenhouse conditions. Values are the mean ± standard error (SE). Different uppercase letters indicate significant differences among the same ecotype under different seasonal conditions at *p* < 0.05, and different lowercase letters indicate significant differences among the different ecotypes under the same seasonal conditions at *p* < 0.05.

The interactions between seasonal variation and ecotypes significantly influenced the ergot concentrations of six *F. sinensis* ecotypes (*p* < 0.05); and the seasonal variation significantly (*p* < 0.05) affected the ergot concentration of six ecotypes of *F. sinensis* (Tables 2, 3). Ecotype had a significant (*p* < 0.05) impact on the ergot concentration only under greenhouse conditions (Table 3). For five ecotypes (41, 57, 84, 99, and 141) of *F. sinensis*, the ergot concentrations peaked in the summer, but lowered in spring and autumn (Figures 5, 6). The ergot concentrations of ecotype 99 and 111 were significantly higher in summer (*p* < 0.05) relative to other four ecotypes (Figure 5). Note that ecotype has no significant effect on the ergot concentration (Figure 6).

## Discussion

We demonstrated that the concentrations of alkaloids produced by the *Epichloë* endophyte-*F. sinensis* associations changed with seasonal variation. There were differences in the trends of the three alkaloids (peramine, lolitrem B, and ergot). We revealed that ecotypes significantly affect some alkaloids under the same seasonal conditions. The concentrations of similar alkaloids were quite different under field and greenhouse conditions.

Many previous studies have shown that grasses without endophyte did not produce alkaloids. It is concluded that the alkaloids tested in our manuscript were only produced by endophyte rather than host plants (Young et al., 2009; Schardl et al., 2013a,b). This study did not detect the three alkoids in the E*-F. siensis* plants through HPLC. Seasonal variation can influence the peramine concentrations produced by the endophyte of *F. sinensis*. A previous study on grazing grassland showed that the peramine concentrations of *L. perenne* displayed a seasonal rhythm with peak concentrations in summer and minimal concentrations in winter (Fuchs et al., 2017). However, our study indicated that the peramine concentrations of six ecotypes of *F. sinensis* decreased from spring to autumn. The *F. sinensis* prefers cooler growth conditions, with a tendency to grow rapidly in spring and autumn but achieves slower growth in summer and winter. The *Epichloë* endophytes rely on their host plants to acquire the desired growth nutrients (Nan and Li, 2004). The growth of *Epichloë* endophyte slows down as the host plants grow slowly. This explains why the concentration of peramine produced by endophyte reduced in summer. In addition, the peramine concentration produced by the host plant is affected by the genotype (Faeth et al., 2002). Peramine is a natural insecticide. High levels of peramine (ranges 54–80 ppm) produced by the *F. sinensis*-endophyte associations would have strong resistance to insect feeding (Tian et al., 2018). The present study showed that the peramine concentrations of six ecotypes of *F. sinensis* bottomed in autumn. This implied that the insect resistance of *F. sinensis*-endophyte associations weakened in autumn. In addition, our results revealed that the ecotype had no significant effect on the peramine concentration produced by *F. sinensis*-endophyte associations under field and greenhouse conditions. We speculated that there were effects of the seasonal conditions instead of ecotypes on the peramine concentration.

Early studies found that ryegrass staggers was caused largely by the presence of lolitrem B (Gallagher et al., 1981; Ball et al., 1995). Endophytic fungal has long been assumed to grow only at the hyphal tip. However, Christensen et al. (2008) showed an intercalary division and extension of fungal tissue, which is connected to enlarging host plant cells, enables the fungal extension at the same rate as the host growth. This study showed that the lolitrem B concentration of *F. sinensis* was higher in autumn than in other seasons corresponding to the plant phenotype, indicating that endophyte grows concurrently with the grass through intercalary division, as postulated by Christensen et al. (2008). Low alkaloid concentrations in young plants indicate alkaloid biosynthesis to be costly (Fuchs et al., 2017). Spring and summer are the growth periods for the *F. sinensis* seedlings. In younger plants, nutrient resources are mainly used for primary metabolism such as plant and endophyte growth, rather than secondary metabolite synthesis (Faeth and Fagan, 2002). The present study also found that the endophytic fungi of the *F. sinensis* produced peramine instead of lolitrem B. This could be caused by chemical complexity and biosynthetic cost difference, which is similar to the plant secondary metabolite synthesis (Nishida, 2002). In addition, temperature rise might increase the alkaloid concentrations (Hennessy et al., 2016).

The present study showed that the interactions between seasonal variation and ecotypes significantly affected the ergot concentrations of six *F. sinensis* ecotypes; and the seasonal variation significantly impacted the ergot concentration of six ecotypes of *F. sinensis*. For five ecotypes (41, 57, 84, 99, and 141) of *F. sinensis*, the ergot concentrations peaked in summer, and declined in spring and autumn. Previous studies suggested that phosphorus had an effect on the ergot alkaloid concentration of *Neotyphodium coenophialum*-infected *Festuca arundinacea* (Malinowski et al., 1998). Our research supported that seasonal change affected the alkaloid concentrations produced by the endophytes of *L. perenne*; the alkaloid concentrations showed a seasonal rhythm with peak concentrations in summer and minimal concentrations in winter across all 3 years (2013–2015) (Fuchs et al., 2017). The short-term cold stress could also affect the ergot alkaloids of *F. sinensis* (Zhou et al., 2015a). Moreover, there were host genotype or endophyte genotype effects on the quantitative differences in the alkaloid concentration of *F. sinensis*. This has been reported by previous studies on different species (Easton et al., 2002; Tian et al., 2013).

## Conclusion

In conclusion, both seasonal variation and ecotypes can affect the alkaloids produced by the *F. sinensis-*endophyte associations (especially true for seasonal variation). Different categories of alkaloids vary differently over a seasonal cycle. In autumn, the peramine concentration of *F. sinensis-*endophyte associations was at the lowest level, but the lolitrem B hit the highest level. The ergot concentration was at the lowest level in summer. Hence, the presence of endophyte is beneficial to *F. sinensis* in natural grasslands.

## Author Contributions

PT, WL, and YK designed experiments. WL, DD, and JW carried out the experiments. CN, MW, ML, and BM analyzed experimental results. WL and WX wrote the manuscript.

### Conflict of Interest Statement

The authors declare that the research was conducted in the absence of any commercial or financial relationships that could be construed as a potential conflict of interest.
